# Diversity Analysis of Intestinal Bifidobacteria in the Hohhot Population

**DOI:** 10.3390/microorganisms12040756

**Published:** 2024-04-09

**Authors:** Shuying Yang, Su Wu, Feiyan Zhao, Zhixin Zhao, Xin Shen, Xia Yu, Meng Zhang, Fang Wen, Zhihong Sun, Bilige Menghe

**Affiliations:** Inner Mongolia Key Laboratory of Dairy Biotechnology and Engineering, Inner Mongolia Agricultural University, Hohhot 010018, China; ying_1226@163.com (S.Y.); wusu6911660@163.com (S.W.); 13190518403@163.com (F.W.);

**Keywords:** *Bifidobacterium*, droplet digital PCR, PacBio Sequel II

## Abstract

(1) Background: *Bifidobacterium* plays a pivotal role within the gut microbiota, significantly affecting host health through its abundance and composition in the intestine. Factors such as age, gender, and living environment exert considerable influence on the gut microbiota, yet scant attention has been directed towards understanding the specific effects of these factors on the *Bifidobacterium* population. Therefore, this study focused on 98 adult fecal samples to conduct absolute and relative quantitative analyses of bifidobacteria. (2) Methods: Using droplet digital PCR and the PacBio Sequel II sequencing platform, this study sought to determine the influence of various factors, including living environment, age, and BMI, on the absolute content and biodiversity of intestinal bifidobacteria. (3) Results: Quantitative results indicated that the bifidobacteria content in the intestinal tract ranged from 10^6^ to 10^9^ CFU/g. Notably, the number of bifidobacteria in the intestinal tract of the school population surpassed that of the off-campus population significantly (*p* = 0.003). Additionally, the group of young people exhibited a significantly higher count of bifidobacteria than the middle-aged and elderly groups (*p* = 0.041). The normal-weight group displayed a significantly higher bifidobacteria count than the obese group (*p* = 0.027). Further analysis of the relative abundance of bifidobacteria under different influencing factors revealed that the living environment emerged as the primary factor affecting the intestinal bifidobacteria structure (*p* = 0.046, R^2^ = 2.411). Moreover, the diversity of bifidobacteria in the intestinal tract of college students surpassed that in the out-of-school population (*p* = 0.034). This was characterized by a notable increase in 11 strains, including *B. longum*, *B. bifidum*, and *B. pseudolongum*, in the intestinal tract of college students, forming a more intricate intestinal bifidobacteria interaction network. (4) Conclusions: In summary, this study elucidated the principal factors affecting intestinal bifidobacteria and delineated their characteristics of intestinal bifidobacteria in diverse populations. By enriching the theory surrounding gut microbiota and health, this study provides essential data support for further investigations into the intricate dynamics of the gut microbiota.

## 1. Introduction

In 2001, Joshua Lederberg introduced the concept of the human microbiome [1]. The primary focus of research revolves around the inheritance and metabolism of microorganisms in various human body sites, including the skin [2], oral cavity [3], vagina [4], and gastrointestinal tract. The objective is to comprehend their impact on host health and pathogenesis. The gut microbiome, a significant subject of human microbiome research, has garnered considerable attention since the proposal of the Human Intestinal Metagenome Initiative (HIMI) during the international conference in 2005 [1]. Over the past two decades, the rapid evolution of sequencing technology has led to an increasing number of reports on the gut microbiome [5]. The gut microbiome encompasses a vast and intricate micro-ecosystem consisting of tens of thousands of bacteria [6], fungi, viruses, and other microorganisms residing in the human gastrointestinal tract. This ecosystem significantly influences human health [7], with imbalances potentially contributing to inflammatory bowel disease [8], immune system disorders, mental health issues, and metabolic diseases. Despite the absence of an accurate definition of a healthy gut microbiome [9], some researchers posit that key attributes include high stability and diversity of the microbiome, robust resistance to stressors such as antibiotics, infection, and immunosuppression, and metabolic pathways beneficial to the human body [9].

*Bifidobacterium*, a Gram-positive bacterium with a high G+C content, was initially discovered by Tissier in 1899 in the feces of breast-fed infants and was subsequently classified under Actinobacteria [10]. It is one of the most common bacteria in the gastrointestinal tract of both humans and animals. Common bifidobacteria species in humans include *B. adolescentis*, *B. angulatum*, *B. bifidum*, *B. breve*, *B. catenulatum*, *B. dentium*, *B. longum*, *B. pseudocatenulatum*, and *B. pseudolongum* [11]. Studies have indicated the significance of bifidobacteria as one of the important floras *maintaining* the balance of microbial communities within the gastrointestinal tract [12]. Disruptions in the gut microbiome are often accompanied by variations in the *Bifidobacterium* levels or species composition [13]. In addition, *Bifidobacterium* facilitates the treatment of diseases such as Irritable Bowel Syndrome (IBS) [14] and tumors [15]. It also enhances cognitive function [15], alleviates anxiety and depression, and promotes the immune system [16,17].

The gut microbiome of adults can be affected by several factors, including age, gender, geographic location, and living or working environment. From early life, a close relationship between the gut microbiome and human body has been established [18]. Early colonization of the gut microbiome not only plays a critical role in infant growth and development but also can further affect the health of all life [19]. Studies have suggested that healthy lactating infants exhibit lower diversity in their gut microbiome than adults. Nevertheless, the content and abundance of bifidobacteria are higher [18,20]. Upon reaching adulthood, the diversity of the gut microbiome typically increases with age, whereas the abundance and diversity of actinomycetes, such as *Bifidobacterium*, begin to decline. This trend is more prevalent in females than in males and tends to stabilize after the age of 40 years. As individuals enter old age, their gut microbiome ages [21]. As the elderly population becomes weak, there is a notable reduction in the α diversity of the gut microbiome [22]. This reduction primarily manifests as a decrease in flora diversity, an increase in pathogenic microorganisms, and a decrease in gut microbiome stability [23]. However, Lorenzo Drago et al. conducted a study comparing the gut microbiome of centenarians and young individuals and discovered that the species and quantity of lactic acid bacteria and bifidobacteria isolated from the intestines of centenarians were similar to those of young individuals [24].

Geography can significantly affect the gut microbiome [25]. One study revealed that closer proximity to geographical environments can contribute to higher similarity in the composition of the gut microbiome of the population. Conversely, for different geographical environments, the gut microbiomes can be different. A study on the gut microbiome of inland and island populations in South Korea suggested that inland populations exhibited higher diversity and richness in their gut microbiome compared to island populations [26]. Similarly, a study involving 314 individuals from 20 provinces and seven ethnic groups in China demonstrated that the gut microbiome of subjects clustered according to geographical location [27]. Furthermore, regional differences are often associated with variations in social systems, economic status, lifestyle, dietary habits, and work environment, all of which can change the gut microbiome [26]. Human history has witnessed three distinct survival stages: foraging, agricultural and rural life, and industrialized urban life, and the human gut microbiome has changed [26]. Notably, the gut microbiome of nomadic hunter/gatherers presents a higher diversity and stability. However, with the development of urbanization and industrialization, as well as the improvement of medical care and health levels, humans have reduced their contact with natural environments such as soil, forests, and livestock, gradually adapting to indoor lifestyles [28]. Simultaneously, the dietary composition predominantly comprises refined high-protein foods. Consequently, the Treponema genus that is beneficial for nutrient absorption among hunter/gatherers and traditional agricultural populations has degraded, creating a favorable environment for the development and functional role of the “Western Microbiome” [29].

Although urbanization offers convenience to individuals’ lives, it also introduces various sources of anxiety and stress. This can affect the composition of the gut microbiome. Chronic stress increases susceptibility to diseases through inflammation. Inflammation plays a pivotal role in the modulation of the intestinal microflora. Numerous reports have demonstrated that high stress levels can cause a reduction in the Simpson index and decreases in Lactobacillus and α-diversity in the intestinal environment [30]. Concurrently, another perspective suggests that the increase in beneficial bacteria, such as *B. infantis* in the intestines, can inhibit the hyperactivity of the Hypothalamic–Pituitary–Adrenal (HPA) axis, reducing norepinephrine secretion and alleviating anxiety and depression [31,32]. The gut microbiome exhibits a high degree of susceptibility and is easy to respond to environmental and host-derived stimuli. It actively regulates its composition and function to coexist and develop harmoniously with the host organism [33]. The alterations in the microbiota can profoundly affect the health of the host, emphasizing the importance of monitoring key factors influencing the gut microbiome.

Currently, the prevalent methods for analyzing gut microbiome quantity include traditional culture techniques and quantitative PCR (q-PCR) for bifidobacterial composition analysis of intestinal samples [34,35]. However, the traditional culture approach is labor-intensive, time-consuming, unable to detect viable but nonculturable (VBNC) cells, and ineffective at distinguishing species or strains with similar colony morphologies [36,37]. It can only detect the total quantity of viable bacteria in a sample. In addition, some studies have found that q-PCR can detect low-content samples, so the combination of q-PCR and specific primers can achieve quantitative detection of bifidobacteria in the intestine. However, q-PCR needs to rely on the standard curve for quantitative detection [38]. Droplet digital PCR (dd-PCR), as a third-generation nucleic acid amplification technology [39], offers a non-cultured quantitative approach capable of directly determining the copy number of target nucleic acid molecules in the sample. It has excellent specificity, high sensitivity, and yields precise results. Advances in high-throughput sequencing technology have provided researchers with more accessible tools. For example, 16S rRNA gene amplicon sequencing is still a standard method for nonculturable studies of microbial diversity and is commonly used to analyze microbial composition in complex samples [40,41]. However, the resolution of 16S rRNA gene sequencing is limited and cannot distinguish between closely related bacterial species [42,43]. In response, our laboratory developed bifidobacteria-specific primers for high-throughput sequencing and dd-PCR quantification, facilitating the investigation of the relative and absolute levels of bifidobacteria at the species level within the intestinal environment.

In summary, the abundance and composition of bifidobacteria in the human gut microbiome is affected by numerous factors. It is necessary to explore the number and composition of bifidobacteria in vivo under different influencing factors. Therefore, this study employed dd-PCR, successfully validated bifidobacteria-specific primer amplification, and third-generation sequencing technology using PacBio Sequel II to analyze the bifidobacterial population and composition within the intestines of adult individuals residing in Hohhot, Inner Mongolia Autonomous Region, China. This study aimed to uncover the impacts of various factors, including living environment, age, and BMI, on bifidobacteria. The results not only established a foundation for future research but also offered potential targets for the prevention, diagnosis, and treatment of specific diseases.

## 2. Materials and Methods

### 2.1. Subject Recruitment and Fecal Sample Collection

**Figure 1:**
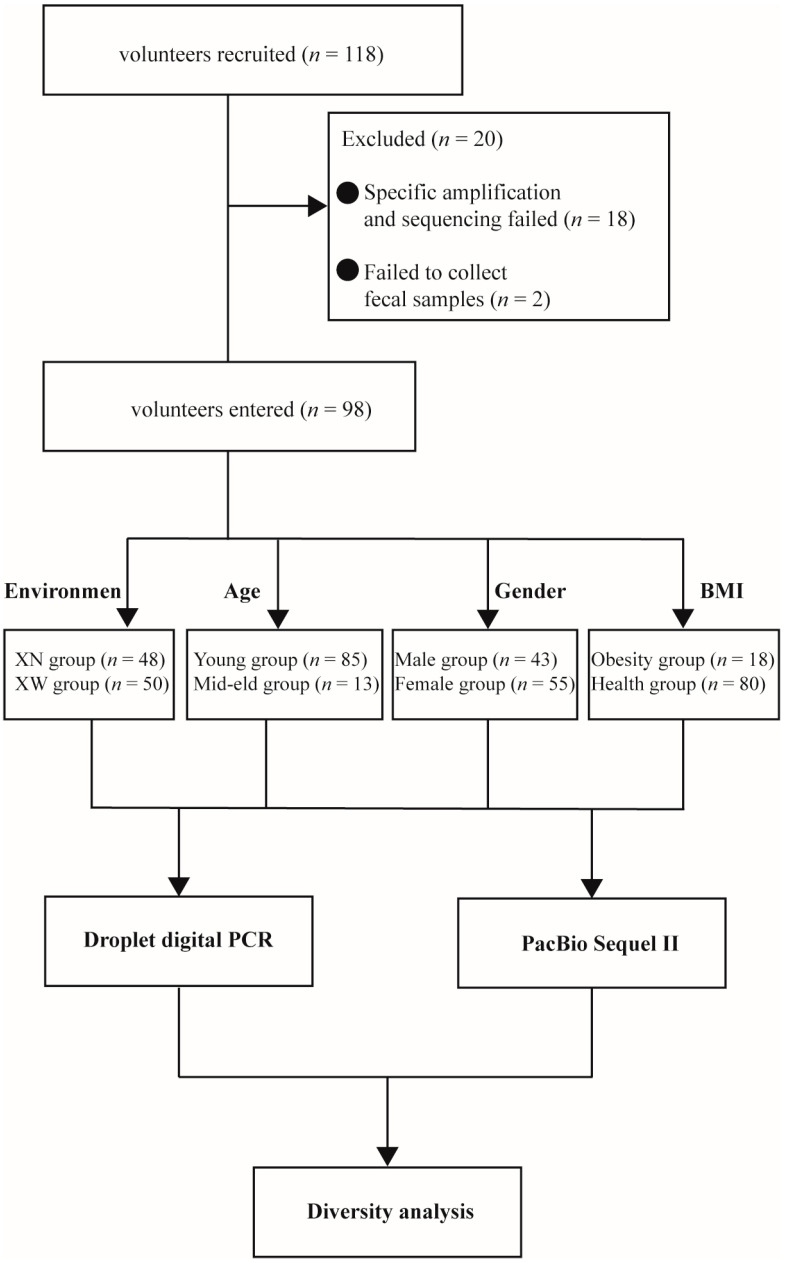
Flow diagram of subject selection.

A total of 118 volunteers were recruited from Hohhot, Inner Mongolia Autonomous Region, China, and 98 fecal samples were collected based on the specific inclusion criteria. Fresh fecal samples were obtained using a tube containing protective solutions and a conventional fecal sampling tube, packaged in ice packs, and transported to the laboratory within 24 h. Subsequently, the fecal samples collected by these two methods were numbered and separated and stored at −80 °C. Fecal samples collected in tubes containing protective solution were used for sequencing, and samples from conventional collection tubes were used for quantitative testing. The inclusion criteria were as follows: (1) participants aged 18 years or older, both male and female, with female volunteers not being pregnant or lactating; (2) no history of severe diarrhea, severe constipation, or gastrointestinal disorders in the preceding month; (3) absence of antibiotic and probiotic use within the last three months; (4) absence of diabetes, hypertension, and hyperlipidemia; and (5) no major illnesses, cognitive impairment, or mental disorders. The excluded criteria were as follows: (1) does not conform to inclusion criteria. (2) poor compliance with the program. Twenty volunteers were excluded from the study based on their willingness to participate and experimental conditions: (1) specific amplification and sequencing failures (*n* = 18); and (2) volunteers who provided informed consent and physical examinations but could not provide fecal samples (*n* = 2). This study received approval from the Ethics Committee of the Affiliated Hospital of Inner Mongolia Medical College (Project Number: NO.KY2020014) and was registered with the Chinese Clinical Trial Registry (http://www.chictr.org.cn/ (accessed on 16 February 2024); registration number: ChiCTR2000038746). Informed consent was obtained from all participants before the commencement of the study. Only partial medical examination data were referenced, and the study did not involve human experiments. The collection of fecal samples posed no foreseeable risks of harm or discomfort to the participants (Figure 1).

### 2.2. DNA Extraction and PacBio Sequel II Sequencing

The total DNA from fecal samples stored in two fecal sampling tubes was extracted using the SPINeasy DNA Spin Kit for Feces (MP Biomedicals, Santa Ana, CA, USA), following the manufacturer’s instructions. *Bifidobacterium*-specific gene sequences were targeted amplified from all genomic DNA samples by polymerase chain reaction (PCR) for PacBio Sequel II sequencing, using the forward sequencing primer (Bif-11F: 5′-AAGAAGAAGGCCACCAAGTAYT-3′) and the reverse sequencing primer (Bif-11R: 5′-GGTAAGAGTCGGACGCTGTGCAATAA-3′) [44]. In the PCR experiment, pure water was used as a template for negative control. The PCR program consisted of the following steps: 95 °C for 1 min, 30 cycles of 95 °C for 1 min, 60 °C for 1 min, and 72 °C for 2 min, with a final extension at 72 °C for 7 min. The amplicons of the *Bifidobacterium*-specific gene were applied to construct DNA libraries using the Pacific Biosciences SMRT Bell™ template prep kit, as previously described [45,46]. DNA that could not be repaired was removed with exonuclease (Pacifc Biosciences, Menlo Park, CA, USA), and the repaired DNA was re-purified to build a high-quality circularized DNA library [47]. The quality of the library was assessed using a Qubit@ 2.0 Fluorometer (Thermo Scientific, Waltham, MA, USA) and the FEMTO Pulse system. According to the instructions, more than 60% of the fragments in the library are within 1600 bp, which is a qualified library. Finally, the library was sequenced using the PacBio Sequel platform. The Sequel II System performs sequencing up to 30 h and features more than eight times the sequencing data output compared to the Sequel System [45].

### 2.3. dd-PCR

Bif-D-7 specific quantitative primers were used for dye method dd-PCR. Pure water was used as negative control in the experiment. The dd-PCR system consisted of the following components: BIF-D7f primer (ATCAATGATTCAGCAGGAAACGC), 0.2 μL; BIF-D7r primer (GTTCTCGTCGAACTTGATGTAGG), 0.2 μL; DNA (or H_2_O), 2 μL; ddH_2_O, 7.6 μL; QX200 dd-PCR EvaGreen SuperMix, 10 μL; and Droplet Generation Oil for EvaGreen, 70 μL [48]. The dd-PCR program was as follows: 95 °C for 10 min, 40 cycles of 94 °C for 30 s and 60 °C for 1 min, followed by cooling at 4 °C for 5 min, an extension step at 90 °C for 5 min, and holding at 12 °C. Upon completion of the PCR cycles, a QX 200TM Droplet Reader was used to read the amplified microdroplets, and the results were calculated using Formula (1).

(1) $$N(\frac{CFU}{g})=\frac{20\times solution\;volume\;(\mu L)\times dilution\;factor\times copy\;number\;(copies/\mu L)}{2\times sample\;mass\;required\;for\;DNA\;extraction\;(g)}$$

### 2.4. Bioinformatics Analyses and Statistical Analyses

According to Yang et al., bioinformatics analysis was conducted on the extracted high-quality sequences using the Quantitative Insights into Microbial Ecology (QIIME1) package (version 1.7). Biological analyses also were performed using the QIIME platform in this study. Sequence proofreading was performed using PyNAST. A two-step UCLUST merging procedure was executed to establish a single sequence set without duplication and operational taxonomic unit (OTU) at 97% similarity thresholds. Chimera Slayer was adopted to eliminate OTUs containing chimeric sequences. Representative sequences of OTUs were aligned with a custom-built *Bifidobacterium* database to annotate the bacterial taxonomy at various levels. Based on the relative abundance of different species, the Upset package in R 4.3.2 software was employed for analyses [49]. Significant differences in alpha diversity indexes between the two groups were identified using Wilcoxon tests. The beta diversity was calculated by using weighted UniFrac distance and displayed using principal coordinate analysis (PCoA) [47]. The generated heatmap demonstrated the distribution of the dominant species (average relative abundance > 0.1%) across different individuals. Subsequently, the Wilcoxon test was employed to assess species differences in bifidobacteria within the intestinal tracts of all volunteers (*p* < 0.05). Furthermore, Spearman rank correlation analysis (|R| > 0.3, *p* < 0.05) was conducted to explore bacterial interaction relationships (https://www.omicstudio.cn/tool (accessed on 25 January 2024)).

## 3. Results

### 3.1. Volunteer Data and Grouping Information

This study comprised 98 volunteers aged 18–64 years, consisting of 43 males and 55 females (Table A1). According to the different environments in which the volunteers live, 48 volunteers are college students living on campus, and the other 52 volunteers are social groups living outside the school. They were divided into the school group, named “XN group” and the off-campus group, named “XW group”. In addition, volunteers were grouped according to age, BMI and gender, and the details are shown in Table 1.

### 3.2. Absolute Quantitative Analysis of Bifidobacterium

**Figure 2:**
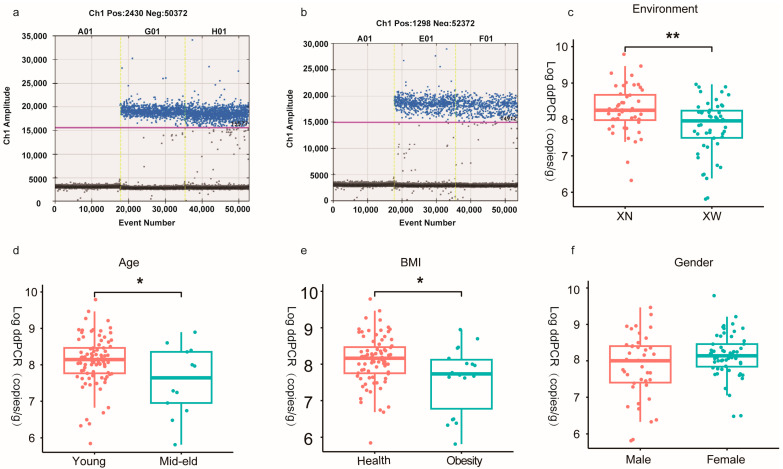
Absolute quantification of bifidobacteria (the LOG value calculated by dd-PCR): (**a**,**b**) Number of droplets distribution of Bifidobacterium in the intestinal tract of college students and social workers; The colored line is the dividing line between positive droplets and negative droplets. The positive droplets are on the line and the negative droplets are off the line. (**c**–**f**) Box plot showing the number of bifidobacteria based on the living environment, age, BMI, and gender. The “*” represents the intensity of significant difference (*, *p* < 0.05; **, *p* < 0.01).

First, the absolute quantification of bifidobacteria in the adult intestine was conducted using dd-PCR (Table A2). The findings revealed that bifidobacteria counts in all volunteers ranged from approximately 10^6^ to 10^9^ CFU/g, with the majority (86.73%) concentrated in the 10^7^ to 10^8^ CFU/g range. A small proportion (13.27%) exhibited counts of either 10^6^ or 10^9^ CFU/g, with the former being exclusive to the social population and the latter limited to college students. Subsequently, the influence of various factors on the content of bifidobacteria in the human intestine was assessed based on the living environment, age, BMI, and gender. The average bifidobacteria biomass in the XN group was (5.1 ± 10) × 10^8^ CFU/g, and the average biomass of bifidobacteria in the XW group was (1.6 ± 2.2) × 10^8^ CFU/g. The XN group of bifidobacteria was significantly larger in the XN group than in the XW group (*p* = 0.0027). Furthermore, the Young group exhibited a significantly higher number of intestinal bifidobacteria than the mid–eld group (*p* = 0.041) in the age analysis. However, the Obesity group displayed a significantly lower bifidobacterial count than the healthy group (*p* = 0.027). Additionally, female individuals demonstrated a higher bifidobacterial content than that in male individuals (*p* = 0.111). In summary, the number of intestinal bifidobacteria was affected by factors such as the living environment, age, BMI, and gender. Among these factors, the living environment appeared to exert a more pronounced effect on intestinal bifidobacterial counts than others (Figure 2).

### 3.3. Diversity Analysis of Bifidobacterium

**Figure 3:**
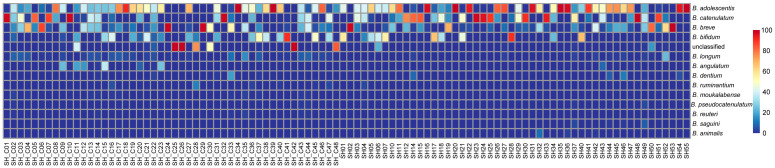
Heat map representing the distribution of bifidobacterial species in each individual.

Subsequently, the effects of various factors on the diversity of bifidobacteria were further investigated. Across all samples, 43 distinct bifidobacterial species were identified, and the dominant species of bifidobacteria (with an average relative content exceeding 0.1%) were screened. The distribution of bifidobacteria within the intestines of different volunteers was thoroughly examined, identifying 14 predominant bifidobacterial species. These species included *B. adolescentis* (37.18%), *B. catenulatum* (23.35%), *B. breve* (16.32%), *B. bifidum* (11.15%), unclassified (6.25%), *B. longum* (1.85%), *B. angulatum* (1.45%), *B. dentium* (0.70%), *B. ruminantium* (0.43%), *B. moukalabense* (0.33%), *B. pseudocatenulatum* (0.26%), *B. reuteri* (0.20%), *B. saguini* (0.19%), and *B. animalis* (0.17%) (Figure 3).

**Figure 4:**
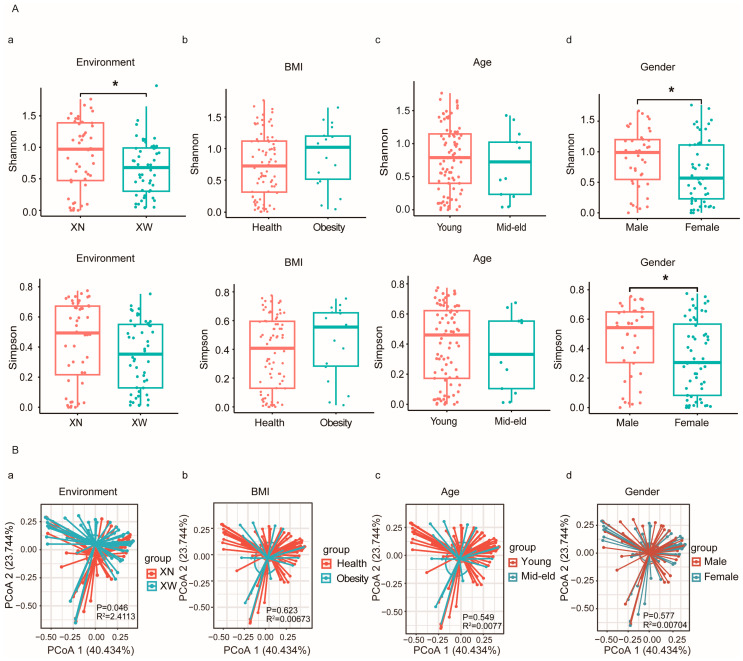
Microbial diversity: (**A**) Shannon index and Simpson index showed the α diversity of each group under different groups: (**a**) is the Shannon index and Simpson index of different living environment; (**b**) is the Shannon index and Simpson index of different BMI; (**c**) is the Shannon index and Simpson index of different ages; (**d**) is the Shannon index and Simpson index of different genders. (**B**) Principal coordinate analysis (PCoA) scores of the two groups under different influencing factors. (* *p* < 0.05); **B** (**a**–**d**) is the PCoA diagram of different living environment, BMI, age and gender groups.

The α diversity index among different groups was analyzed, revealing significant effects of living environment and gender on the diversity of intestinal bifidobacteria. Specifically, the Shannon index showed a significantly higher diversity of intestinal bifidobacteria in the XN group than in the XW group (*p* = 0.034). Furthermore, the Shannon index (*p* = 0.029) and Simpson index (*p* = 0.02) indicated a significantly higher diversity of bifidobacteria among male volunteers than among female volunteers. Although the Young group exhibited higher diversity in intestinal bifidobacteria than the mid–eld group, this difference was not statistically significant. Notably, the diversity of bifidobacteria in the intestines of the obese individuals exceeded that of the normal group. Subsequent application of PCoA revealed distinct separation trends in bifidobacteria within the intestines of individuals living in different environments (*p* = 0.046, R^2^ = 2.4113), and other groups did not display noticeable separation trends. Hence, when considering factors such as age, BMI, and gender, the living environment predominantly affected the diversity of intestinal bifidobacteria in adults. Specifically, the α diversity in the XN group was significantly higher than that in the XW group. Combined with the quantitative and diversity results, the living environment played a pivotal role in affecting both the quantity and structural composition of intestinal bifidobacteria compared to age, BMI, and gender. Therefore, subsequent analysis was based on the impact of the living environment on bifidobacteria (Figure 4).

### 3.4. Analysis of Differential Bacteria

**Figure 5:**
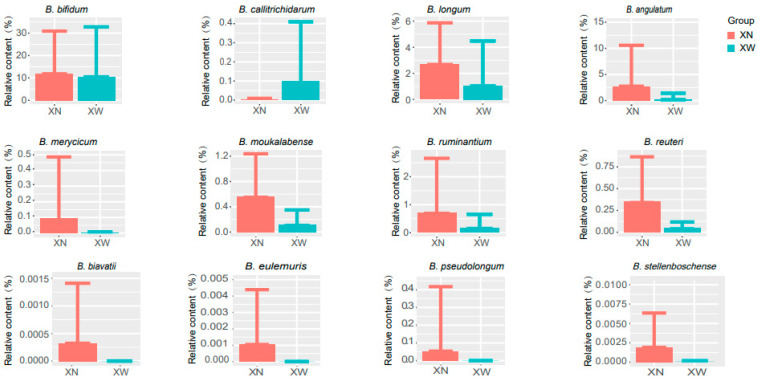
Based on the annotated relative abundance of bifidobacteria, species with significant differences in intestinal bifidobacteria between the two groups of people in different living environments were selected (*p* < 0.05), and the content of bifidobacteria in each group is represented by a histogram.

The Wilcoxon test was applied to analyze the differential species of intestinal bifidobacteria, identifying a total of 12 strains with significant differences (*p* < 0.05). These strains included *B. angulatum*, *B. moukalabense*, *B. stellenboschense*, *B. reuteri*, *B. merycicum*, *B. longum*, *B. ruminantium*, *B. eulemuris*, *B. bifidum*, *B. pseudolongum*, *B. biavatii*, and *B. callitrichidarum*. Noteworthy findings included a significantly higher content of *B. callitrichidarum* in the XW group than in the XN group (XW vs. XN: 0.100% ± 0.310% vs. 0.003% ± 0.006%). In the XN group, the following species were found to have higher levels compared to the XW group: *B. angulatum* (XN vs. XW: 2.763% ± 7.816% vs. 0.190% ± 1.211%), *B. moukalabense* (XN vs. XW: 0.100% ± 0.310% vs. 0.100% ± 0.515%), *B. stellenboschense* (XN vs. XW: 0.002% ± 0.004% vs. 0% ± 0%), *B. reuteri* (XN vs. XW: 0.351% ± 0.004% vs. 0% ± 0%), *B. merycicum* (XN vs. XW: 0.093% ± 0.393% vs. 0.0011% ± 0.007%), *B. longum* (XN vs. XW: 2.688% ± 3.180% vs. 1.045% ± 3.431%), *B. ruminantium* (XN vs. XW: 0.701% ± 1.956% vs. 0.161% ± 0.495%), *B. eulemuris* (XN vs. XW: 0.001% ± 0.003% vs. 0% ± 0%), *B. bifidum* (XN vs. XW: 11.839% ± 19.041% vs. 10.490% ± 22.273%), *B. pseudolongum* (XN vs. XW: 0.054% ± 0.364% vs. 0% ± 0%), *B. biavatii* (XN vs. XW: 0.0003% ± 0.0011% vs. 0% ± 0%), and *B. callitrichidarum* (XN vs. XW: 0.003% ± 0.006% vs. 0.010% ± 0.310%). Among them, *B. biavatii*, *B. eulemuris*, *B. pseudolongum*, and *B. stellenboschense* were exclusively present in the XN group (Figure 5).

### 3.5. Analysis of Interaction Relationships of Flora Network

**Figure 6:**
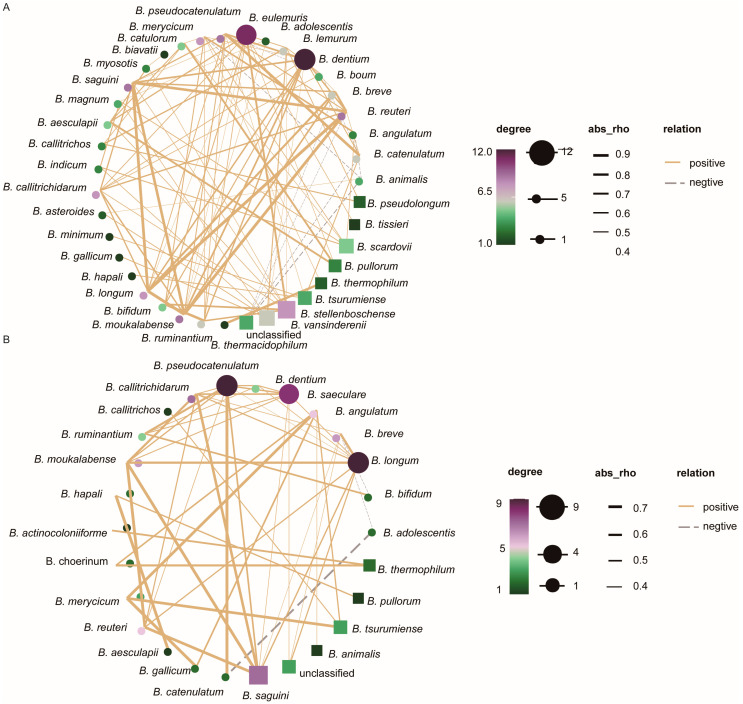
Species co-occurrence network of *Bifidobacterium* in different living environments. Solid and dotted lines represent positive correlation and negative correlation, respectively. Thick and thin lines represent the corresponding correlation strength, respectively (|R| > 0.3, *p* < 0.05). (**A**) represents the network interaction diagram of bifidobacteria in the intestinal tract of college students (XN group); (**B**) represents the network interaction diagram of bifidobacteria in the intestinal tract of the social population (XW group).

Finally, Spearman correlation analysis was conducted to examine the impacts of environmental factors on the network interactions of intestinal bifidobacteria. The correlation strength was defined as follows, according to the report by Zhao et al. [50] and Silva et al. [51]: very strong (|R| ≥ 0.8), strong (0.6 ≤ |R| < 0.8), moderate (0.5 ≤ |R| < 0.6), and weak (0.3 < |R| < 0.5). The analysis revealed that the network relationships among intestinal bifidobacteria in the XN group were denser than those in the XW group. Notably, there were 49 pairs of species interactions, including two weak negative correlations, such as *B. breve* and *B. adolescentis* (R = −0.31), and *B. breve* and *B. bifidum* (R = −0.31). Additionally, a moderate negative correlation was observed between *B. adolescentis* and *B. catenulatum* (R = −0.55). Furthermore, there were 27 weak positive correlations, including *B. breve* and *B. calitrichidarum* (R = 0.34), and *B. angulatum* and *B. animalis* (R = 0.32). Moreover, eight moderate positive correlations were observed, including *B. ruminantium* and *B. pseudocatenulatum* (R = 0.59), and *B. actinocoloniiforme* and *B.thermophilum* (R = 0.54). Finally, 11 strong positive correlations were observed, including *B. catenulatum* and *B. pseudocatenulatum* (R = 0.72), and *B. longum* and *B. breve* (R = 0.61). In contrast, the XN group exhibited ninety-five pairs of species interactions, encompassing eight weak negative correlations, such as *B. boum* and *B. pseudocatenulatum* (R = −0.30), and *B. angulatum* and *B. angulatum* (R = −0.33). Furthermore, 55 weak positive correlations were identified, e.g., *B. bifidum* and *B. dentium* (R = 0.49), and *B. longum* and *B. pseudocatenulatum* (R = 0.43). Additionally, moderate positive correlations were evident between *B. dentium* and *B. longum* (R = 0.58), and *B. breve* and *B. saguini* (R = 0.58). There were 11 strong positive correlations, such as *B. dentium* and *B. pseudocatenulatum* (R = 0.66), and *B. catulorum* and *B. pullorum* (R = 0.68). Finally, nine strong positive correlations were observed: *B. longum* and *B. moukalabense* (R = 0.96), *B. saguini* and *B. reuteri* (R = 0.87), and *B. catenulatum* and *B. pseudocatenulatum* (R = 0.89) (Figure 6).

## 4. Discussion

The gut microbiota plays a vital role in overall health, and bifidobacteria residing in the intestines are recognized as indicators of health [52]. The structure, diversity, and composition of the gut microbiota are affected by several factors including age, geographical location, and dietary habits. In this study, bifidobacteria-specific primers were utilized combined with the PacBio sequencing platform to elucidate the differences in the abundance, diversity, and composition of bifidobacteria among volunteers residing in diverse environmental settings. 

In addition to gender and age, BMI and living environment exert significant impacts on the abundance of bifidobacteria in the intestines. However, these findings differ from those of Zhang et al., which emphasized that gender was the most influencing factor affecting gut microbiota composition [53]. This can be attributed to two key reasons. First, there was regional variation among volunteers. Zhang’s study focused on individuals in the Pinggu district of Beijing, whereas our research focused on volunteers in Inner Mongolia. Although these regions are geographically close, their dietary and lifestyle disparities are considerable. Second, the adopted sequencing methods were different. Zhang primarily employed metagenomic sequencing to obtain the relative abundance of *Bifidobacterium* in the intestines, whereas this study adopted *Bifidobacterium* genus-specific primers combined with dd-PCR to obtain the absolute content of *Bifidobacterium* in the intestines. Therefore, this study complemented the limitations of previous studies. Additionally, the results were consistent with those of previous studies [54], demonstrating that the absolute content of bifidobacteria in the intestines of young individuals significantly surpassed that in the elderly group. Furthermore, college students exhibited a notably higher absolute content of intestinal bifidobacteria than social workers. Moreover, individuals with normal BMI exhibited higher bifidobacterial content than that of obese people. Bifidobacteria colonized vigorously in early life, constituting approximately 60–70% of the total intestinal flora. This population gradually declined with age, decreasing to 5% or less in the intestines of the elderly population. However, a significant increase in the long-lived population was evident [54]. This study similarly observed this phenomenon, although the oldest volunteer in our study was 64 years old, categorizing them as *Bifidobacterium* long-lived elderly individuals in this study. Additionally, bifidobacteria in the intestines have long been regarded as markers of good health, and a high BMI can be associated with various diseases [55], including cardiovascular, digestive, and respiratory diseases. Significantly reduced bifidobacterial content has been observed in patients or disease models related to these conditions [56,57,58]. College students predominantly face academic stress, whereas social workers contend with life and familial and occupational pressures, leading to differences in dietary and lifestyle patterns between both groups. Therefore, we hypothesized that the variance in bifidobacterial content may be linked to the higher stress and anxiety levels experienced by the social worker population compared to college students, in addition to divergent dietary and lifestyle habits.

Subsequently, we conducted a *Bifidobacterium*-specific analysis to examine the differences in *Bifidobacterium* populations among different groups. Our findings revealed that compared to other factors, the living environment exerted the most significant impact on intestinal bifidobacteria. Notably, the diversity of intestinal bifidobacteria was substantially increased in college students, particularly with a marked increase in the content of *B. longum*, *B. bifidum*, and *B. pseudolongum* in the intestines of the XN group. An increase in gut microbiota diversity is widely associated with better health, and bifidobacteria in the intestine are recognized to be integral to overall well-being [59]. A deficiency in bifidobacteria has been directly linked to anxiety and stress [16,60]. In an RCT experiment, we observed that *B. longum* supplementation regulated neural activity during rest, enhanced vitality, and reduced mental fatigue in volunteers, thereby mitigating negative emotions [16]. Furthermore, *B. pseudolongum*, a producer of acetic acid [61], and chronic stress-induced diseases can be alleviated by modulating immune cells through acetic acid supplementation [62]. Additionally, supplementation with *B. bifidum* strains has been shown to enhance cognitive flexibility in the elderly, increase stress scores, and increase serum levels of brain-derived neurotrophic factor (BDNF) [63]. Simultaneously, studies have indicated that *B. longum* is prevalent in the intestinal tracts of adults, and its metabolism adapts to specific carbohydrate components in the host. The observed change in gut microbiota composition can further verify our hypothesis that stress and anxiety levels in college students were lower than those in social workers. This difference in the absolute and relative abundance of intestinal bifidobacteria between the two groups can be attributed to this trend. In addition, *B. longum* exhibited characteristics related to the interaction between the gut microbiota and host in the intestines. Our observations revealed that the interaction network of bifidobacteria in the intestines of college students, where *B. longum* content was significantly elevated, was more tightly connected, which was consistent with prior research [64]. Furthermore, we identified a significant positive correlation between *B. longum* in the intestines of college students, and between *B. dentium* and *B. moukalabense*., frequently isolated from healthy infant stools, which stimulated intestinal serotonin production and produced the neurotransmitter γ-aminobutyric acid, thereby regulating the gut–brain axis in gnotobiotic mice. Notably, *B. moukalabense* was significantly more abundant in the college student group than in the social worker group. Hence, we hypothesized that specific bifidobacteria, such as *B. longum*, in the gut can affect stress and anxiety regulation by modulating microbial communities [65].

## 5. Conclusions

In summary, this study involved the sequencing of absolute bifidobacterial content and diversity within distinct population groups. Our findings indicated that compared to other factors, living environment and occupational type were the most influential factors affecting intestinal bifidobacteria. Specifically, we observed a substantial increase in both the content and diversity of intestinal bifidobacteria among college students compared to the social population. Furthermore, the interaction network of bifidobacteria displayed greater connectivity, particularly involving flora such as *B. longum*, *B. moukalabense*, and *B. bifidum*, all of which played roles in the regulation of stress and anxiety.

## Figures and Tables

**Table 1:** The volunteer grouping information.

Group	Living Environment		Age		Gender		BMI	
Fundamentum divisions	Differences in living environment		18 ≤ Age ≤ 39, Young; Age > 39, Middle aged and elderly people.		----		BMI > 28, Obesity; BMI < 28, Health.	
Group name	XN	XW	Young	Mid–eld	Male	Female	Obesity	Health
Number (*n*)	48	50	85	13	43	55	18	80

## Data Availability

The raw sequence data reported in this paper have been deposited in the Genome Sequence Archive (Genomics, Proteomics & Bioinformatics 2021) in National Genomics Data Center (Nucleic Acids Res 2022), China National Center for Bioinformation/Beijing Institute of Genomics, Chinese Academy of Sciences (GSA: CRA015535) that are publicly accessible at https://ngdc.cncb.ac.cn/gsa (accessed on 25 March 2024).

## Appendix A

**Table A1:** Information of volunteers.

Sample	Age	Gender	Height (m)	Weight (kg)	BMI
SH_C01	18	Female	1.7	78	26.98962
SH_C02	18	Male	1.76	57	18.40134
SH_C03	19	Male	1.8	95	29.32099
SH_C04	19	Female	1.6	65	25.39063
SH_C05	19	Female	1.58	51	20.42942
SH_C06	19	Female	1.66	51	18.50777
SH_C07	19	Female	1.75	60	19.59184
SH_C08	20	Female	1.66	60	21.77384
SH_C09	20	Male	1.83	65	19.40936
SH_C10	20	Male	1.78	60	18.937
SH_C11	20	Female	1.65	55	20.20202
SH_C12	20	Female	1.75	80	26.12245
SH_C13	20	Female	1.65	52	19.10009
SH_C14	20	Male	1.71	55	18.80921
SH_C15	20	Male	1.85	106	30.97151
SH_C16	20	Male	1.75	63	20.57143
SH_C17	20	Male	1.86	70	20.23355
SH_C18	20	Male	1.78	70	22.09317
SH_C19	20	Male	1.7	60	20.76125
SH_C20	20	Male	1.8	75	23.14815
SH_C21	20	Female	1.65	47	17.26354
SH_C22	20	Female	1.7	60	20.76125
SH_C23	20	Female	1.58	50	20.02884
SH_C24	20	Male	1.84	80	23.62949
SH_C25	20	Female	1.64	52	19.33373
SH_C26	20	Female	1.65	62	22.77319
SH_C27	20	Female	1.58	48	19.22769
SH_C28	20	Male	1.84	107	31.60444
SH_C29	20	Female	1.64	53	19.70553
SH_C30	20	Male	1.8	60	18.51852
SH_C31	21	Male	1.85	125	36.52301
SH_C32	21	Male	1.78	65	20.51509
SH_C33	21	Female	1.73	70	23.38869
SH_C34	21	Female	1.58	54	21.63115
SH_C35	21	Male	1.79	60	18.72601
SH_C36	22	Male	1.78	60	18.937
SH_C37	22	Female	1.58	45	18.02596
SH_C38	22	Female	1.66	68	24.67702
SH_C39	22	Female	1.61	55	21.21832
SH_C40	22	Male	1.9	72	19.9446
SH_C41	22	Male	1.78	115	36.29592
SH_C42	22	Male	1.8	65	20.06173
SH_C43	22	Female	1.72	57	19.26717
SH_C44	22	Male	1.85	65	18.99196
SH_C45	22	Male	1.75	66	21.55102
SH_C46	23	Male	1.9	105	29.08587
SH_C47	23	Male	1.81	100	30.5241
SH_C48	23	Female	1.69	83	29.06061
SH01	53	Male	1.75	72.5	23.67347
SH02	25	Male	1.76	87.6	28.27996
SH03	25	Female	1.62	48.2	18.3661
SH04	41	Female	1.5	58	25.77778
SH05	31	Female	1.7	70	24.22145
SH06	37	Female	1.61	58.9	22.72289
SH07	25	Male	1.79	81.2	25.34253
SH10	27	Male	1.75	76.2	24.88163
SH11	33	Male	1.77	83.6	26.68454
SH12	33	Male	1.75	84.5	27.59184
SH14	55	Female	1.57	59.5	24.13891
SH15	53	Female	1.59	50	19.7777
SH16	38	Male	1.71	71.7	24.52037
SH17	38	Female	1.62	69.9	26.63466
SH18	26	Male	1.85	124.2	36.28926
SH19	31	Female	1.56	69	28.35306
SH20	48	Female	1.54	71	29.93759
SH21	33	Male	1.68	84.7	30.00992
SH22	24	Female	1.68	60	21.2585
SH23	23	Female	1.57	47	19.06771
SH24	23	Female	1.68	53	18.77834
SH25	28	Female	1.65	57.7	21.19376
SH26	26	Female	1.71	81.3	27.80343
SH27	27	Female	1.57	41.5	16.83638
SH28	26	Female	1.58	50.6	20.26919
SH29	24	Male	1.9	68.8	19.05817
SH30	28	Female	1.57	57.6	23.36809
SH31	27	Female	1.62	55.5	21.14769
SH32	25	Male	1.79	65.8	20.53619
SH33	29	Female	1.6	66.2	25.85938
SH34	35	Male	1.78	94.9	29.95203
SH35	36	Female	1.59	62	24.52435
SH36	61	Female	1.59	79.8	31.56521
SH37	53	Male	1.72	82	27.71769
SH40	25	Female	1.64	47.6	17.6978
SH41	24	Male	1.85	74.1	21.65084
SH42	63	Male	1.61	63.6	24.53609
SH43	23	Female	1.7	62.5	21.6263
SH44	24	Female	1.65	72.1	26.48301
SH45	37	Female	1.61	59.6	22.99294
SH46	64	Female	1.57	69.6	28.23644
SH47	37	Female	1.58	54.2	21.71126
SH48	38	Female	1.66	61	22.13674
SH49	33	Female	1.56	74.6	30.65417
SH50	25	Female	1.64	51.1	18.99911
SH51	59	Male	1.65	68.4	25.12397
SH52	64	Male	1.72	90	30.42185
SH53	24	Female	1.71	58.2	19.90356
SH54	64	Male	1.62	64.4	24.53894
SH55	62	Female	1.58	57.7	23.11328

**Table A2:** Details of dd-PCR.

Sample	Fecal Quantity (g)	Return Solution Volume (μL)	Dilution Multiple	Copy Number (Copies/μL)	N (CFU/g)
SH_C01	0.12	100	200	117	1.95 × 10^8^
SH_C02	0.14	100	100	144	1.03 × 10^8^
SH_C03	0.13	100	100	1092.91	8.41 × 10^8^
SH_C04	0.1	100	1000	18.31	1.83 × 10^8^
SH_C05	0.1	100	100	54.7	5.47 × 10^7^
SH_C06	0.12	100	100	599	4.99 × 10^8^
SH_C07	0.12	100	1000	740	6.17 × 10^9^
SH_C08	0.15	100	1000	17.5	1.17 × 10^8^
SH_C09	0.13	100	1000	13.01	1.00 × 10^8^
SH_C10	0.1	100	1000	91.43	9.14 × 10^8^
SH_C11	0.11	100	100	1139	1.04 × 10^9^
SH_C12	0.26	100	200	374	2.88 × 10^8^
SH_C13	0.13	100	100	1110.91	8.55 × 10^8^
SH_C14	0.11	100	1000	28.11	2.56 × 10^8^
SH_C15	0.11	100	100	2.33	2.12 × 10^6^
SH_C16	0.18	100	200	6	6.67 × 10^6^
SH_C17	0.13	100	1000	242.93	1.87 × 10^9^
SH_C18	0.23	100	1000	31.43	1.37 × 10^8^
SH_C19	0.11	100	1000	321.93	2.93 × 10^9^
SH_C20	0.1	100	1000	31.63	3.16 × 10^8^
SH_C21	0.09	100	1000	147	1.63 × 10^9^
SH_C22	0.12	100	100	559	4.66 × 10^8^
SH_C23	0.12	100	100	774	6.45 × 10^8^
SH_C24	0.12	100	1000	6.83	5.69 × 10^7^
SH_C25	0.17	100	100	178.93	1.05 × 10^8^
SH_C26	0.19	100	100	83.7	4.41 × 10^7^
SH_C27	0.14	100	100	268	1.91 × 10^8^
SH_C28	0.11	100	1000	15.83	1.44 × 10^8^
SH_C29	0.19	100	1000	11.1	5.84 × 10^7^
SH_C30	0.13	100	1000	115.73	8.90 × 10^8^
SH_C31	0.11	100	100	837	7.61 × 10^8^
SH_C32	0.12	100	200	253	4.22 × 10^8^
SH_C33	0.12	100	100	219	1.83 × 10^8^
SH_C34	0.25	100	1000	43.93	1.76 × 10^8^
SH_C35	0.15	100	100	164	1.09 × 10^8^
SH_C36	0.19	100	1000	44.6	2.35 × 10^8^
SH_C37	0.12	100	1000	34.91	2.91 × 10^8^
SH_C38	0.15	100	100	126	8.40 × 10^7^
SH_C39	0.12	100	1000	16.71	1.39 × 10^8^
SH_C40	0.18	100	100	182	1.01 × 10^8^
SH_C41	0.1	100	100	41.8	4.18 × 10^7^
SH_C42	0.12	100	200	119.4	1.99 × 10^8^
SH_C43	0.19	100	100	192	1.01 × 10^8^
SH_C44	0.2	100	1000	5.63	2.82 × 10^7^
SH_C45	0.14	100	100	34.03	2.43 × 10^7^
SH_C46	0.12	100	100	195	1.63 × 10^8^
SH_C47	0.14	100	100	42.33	3.02 × 10^7^
SH_C48	0.2	100	100	128.93	6.45 × 10^7^
SH01	0.27	100	1000	5.2	1.93 × 10^7^
SH02	0.13	100	1000	6.1	4.70 × 10^7^
SH03	0.11	200	1000	50.7	9.22 × 10^8^
SH04	0.1	200	100	21.9	4.38 × 10^7^
SH05	0.1	200	100	16.4	3.28 × 10^7^
SH06	0.12	200	100	95.4	1.59 × 10^8^
SH07	0.13	200	100	20	3.08 × 10^7^
SH10	0.18	200	1000	13.7	1.52 × 10^8^
SH11	0.15	100	1000	10.3	6.87 × 10^7^
SH12	0.11	100	100	5.3	4.82 × 10^6^
SH14	0.13	100	100	1022	7.86 × 10^8^
SH15	0.17	100	100	29.6	1.74 × 10^7^
SH16	0.18	100	100	38.7	2.15 × 10^7^
SH17	0.12	100	100	13.5	1.13 × 10^7^
SH18	0.12	100	100	331	2.76 × 10^8^
SH19	0.17	100	1000	0.53	3.12 × 10^6^
SH20	0.11	100	100	110.2	1.00 × 10^8^
SH21	0.16	100	100	80.6	5.04 × 10^7^
SH22	0.1	100	500	47	2.35 × 10^8^
SH23	0.29	100	500	23.4	4.03 × 10^7^
SH24	0.11	100	500	19.9	9.05 × 10^7^
SH25	0.13	100	500	46.4	1.78 × 10^8^
SH26	0.17	100	100	209	1.23 × 10^8^
SH27	0.13	100	100	458	3.52 × 10^8^
SH28	0.13	100	500	40.1	1.54 × 10^8^
SH29	0.11	100	500	10.4	4.73 × 10^7^
SH30	0.12	100	500	28.1	1.17 × 10^8^
SH31	0.08	100	500	26.2	1.64 × 10^8^
SH32	0.1	100	500	0.14	7.00 × 10^5^
SH33	0.09	100	500	20.8	1.16 × 10^8^
SH34	0.15	100	200	1.8	2.40 × 10^6^
SH35	0.1	100	500	44	2.20 × 10^8^
SH36	0.1	100	500	0.6	3.00 × 10^6^
SH37	0.12	100	100	272	2.27 × 10^8^
SH40	0.11	100	100	173	1.57 × 10^8^
SH41	0.11	100	500	126.7	5.76 × 10^8^
SH42	0.14	100	500	2.5	8.93 × 10^6^
SH43	0.12	100	100	149	1.24 × 10^8^
SH44	0.08	100	100	381	4.76 × 10^8^
SH45	0.1	100	100	60.6	6.06 × 10^7^
SH46	0.18	100	500	33.3	9.25 × 10^7^
SH47	0.1	100	500	116.5	5.83 × 10^8^
SH48	0.06	100	500	93.2	7.77 × 10^8^
SH49	0.14	100	100	146	1.04 × 10^8^
SH50	0.11	100	500	9.3	4.23 × 10^7^
SH51	0.2	100	500	2.2	5.50 × 10^6^
SH52	0.1	100	500	0.13	6.50 × 10^5^
SH53	0.2	100	100	142	7.10 × 10^7^
SH54	0.11	100	500	54.8	2.49 × 10^8^
SH55	0.17	100	500	13.6	4.00 × 10^7^
